# On the effect of international human migration on nations’ abilities to attain CO_2_ emission-reduction targets

**DOI:** 10.1371/journal.pone.0258087

**Published:** 2021-10-04

**Authors:** Douglas W. Morris

**Affiliations:** Department of Biology, Lakehead University, Thunder Bay, ON, Canada; Institute for Advanced Sustainability Studies, GERMANY

## Abstract

I merge publicly available data on CO_2_ emissions, with patterns of human movement, to analyze the anticipated effects of human migration on the abilities of nations to attain 2030 UNFCCC CO_2_ emission targets. I do so at both global (175 countries) and national (Canada and the USA) scales. The analyses reveal that mean per capita CO_2_ emissions are nearly three times higher in countries with net immigration than in countries with net emigration. Those differences project a cumulative migration-induced annual increase in global emissions of approximately 1.7 billion tonnes. For Canada and the United States, the projected total emissions attributable to migration from 2021 to 2030 vary between 0.7 and 0.9 billion tonnes. Although staggering, the annual and total emissions represent a small fraction of current global emissions totalling 36 billion tonnes per annum. Even so, the projected decadal immigration of nearly 4 million humans to Canada, and 10 million to the USA, represent significant additional challenges in reducing CO_2_ emissions. The challenges pale in comparison with poor nations that are minor contributors to climate change. Such nations face the incomprehensible burden of improving the quality of their citizens’ lives without increasing global CO_2_ emissions. National and international strategies aimed at lowering emissions must thus acknowledge, and cooperatively address, consumptive inequities and expected increases in human population size and migration.

## Introduction

The 2015 COP21 United Nations Framework Convention on Climate Change (UNFCCC) called for its signatory nations to limit the global “temperature increase to 1.5°C above pre-industrial levels” [1]. In order to do so while maintaining current population sizes, nations must reduce their per capita emissions of CO_2_ and other greenhouse gases. Any increase in population size and its associated demand for energy, whether through births or net immigration, will necessitate an even greater effort to reduce CO_2_ emissions. Reductions in energy demand, as may occur through net emigration, will allow such nations greater opportunity to reach their COP21 commitments. It is thus prudent to evaluate the degree to which population changes induced by international migration modify nations’ abilities to meet their CO_2_ emission targets.

The interactions among human migration, climate change, and CO_2_ emissions are similarly recognized as significant global concerns in the United Nations General Assembly’s 2018 adoption of two important compacts on human movements: the Global Compact on Refugees (GCR; [2]) and the Global Compact for Migration (GCM; [3]. The Global Compact for Refugees includes climate and environmental degradation as indirect influences on refugee movements. The Global Compact for Migration is more explicit in its aims to ensure “safe, orderly and regular migration” while providing “objective, evidence-based, clear information about the benefits and challenges of migration”. Similarly, the International Organization for Migration recognizes “the need to better integrate migration into global climate and environmental mechanisms, and for climate change mechanisms to incorporate human mobility aspects” [4, p.268]. The two Compacts highlight the pressing need for an evidence-based evaluation of the potential effect of human migration on climate change and nations’ abilities to meet their COP21 targets.

Such an evaluation is also necessary in order to meet the GCM’s objective of minimizing “the adverse drivers and structural factors that compel people to leave their country of origin” including “climate change mitigation and adaptation” and the “adverse effects of climate change” ([3, objective 2, 18b, h, i]. The importance of such an analysis is underscored by a recent special issue of International Migration, devoted to the GCR and GCM, that included no detailed “connection between environmental change and migration or refugee movements” [5, p. 12].

Virtually all strategies to achieve the UNFCCC target include major reductions in energy use and replacing fossil fuels with ‘green’ options. Each will have substantial economic, political, social and ecological effects. Technological efficiencies, although important avenues for reducing CO_2_ emissions, interact with both population size and consumption (e.g., through the famous IPAT equation and its predecessors and descendants [6–8]). Technology cannot support an ever-expanding human population on a world shared with millions of other species. Indeed, the most effective (and provocative [9–12]) individual mechanism to reduce CO_2_ equivalent energy use is simply for parents to produce one less child [13,14]. Although the statement applies to parents in all nations, the largest individual impact is in those nations with high CO_2_ emissions. Others have argued that so-called population engineering is not only necessary to address climate change, but that it is morally justifiable, and when merged with migration, economically valuable to do so [15,16].

One reason why reduced reproduction has such a large effect on reducing emissions is that a full accounting of an individual’s carbon legacy, like that of compound interest, must integrate current with future energy use over the otherwise normal lifetime of the individual and appropriately discounted descendants [13]. Population dynamics has only three processes—births, deaths and migration. The central issue is thus not so much about producing fewer children, but is instead about any process that limits human population growth in high CO_2_ emitting nations.

Immigration, except during the 2020–2021 COVID-19 pandemic, is higher than ever [17] and represents a substantial contributor to population growth in many of the World’s richest and high carbon-emitting nations, and especially so in Canada and the USA. On a global scale, the USA has the highest immigrant population (50.7 million in 2019), and the highest number of immigrants between 1995 and 2020 [17]. Canada’s immigrant total is less (8 million) but Canada ranks 5^th^ in the number of recent immigrants, and its population is composed of a much higher percentage of immigrants (21% vs 15% in the USA). The rate of recent immigration into Canada is also much greater than in the USA (estimated net migration rate for 5 years prior to 2020 = 6.6 per thousand persons versus 2.9 per thousand persons in the USA; [17]). Both countries are expected to retain their status with high numbers of immigrants (USA) and high immigration rates (Canada) even if global population growth declines this century [18]. These expectations reflect the long-term 1995–2010 trend of more-or-less constant 5-year global migration flows of 0.6% of all humans [19]. The trend accentuates the urgency of exploring potential impacts of human migration on CO_2_ emissions, the ability of nations to meet UNFCCC targets, and especially so in high immigration nations such as Canada and the USA.

Canada pledged a 30% reduction in 2005 greenhouse gas emissions by 2030. At the end of 2019, Canada set an even more ambitious target of net-zero emissions by 2050 [20], and in April 2021, Canada’s Prime Minister announced at the Leaders’ Summit on Climate that Canada will enhance its 2030 reduction target to 40–45% below 2005 levels [21]. The USA aimed for a more aggressive 26–28% reduction by 2025, then submitted formal notification of its withdrawal from the Paris Agreement on 4 November 2019 [22]. President Biden’s January 2021 Executive Order [23] committed the USA to rejoin the Paris Agreement and host the Leaders’ Climate Summit as part of its contribution to COP26. The President doubled-down at the summit by announcing that the U.S. target for 2030 would achieve a 50–52% reduction from 2005 levels and attain net zero emissions by 2050 [24].

Human migration is often characterized as rational opportunity-seeking behavior constrained by multifarious limitations on human movement (e.g., [25,26] provides an evolutionary explanation). Such choice-based migration does not include the large numbers of displaced humans increasingly connected with climate change and emergent policy initiatives [27]. Whether so-called ‘climate migration’ [28] represents a major future source of human movement can only be assessed in the context of reliable data [29,30]. Such an analysis must also include an objective assessment of the likely consequences of human migration on climate change itself, as well as its potential feedback compelling people to leave their country of origin (Objective 2 in the Global Compact for Safe, Orderly and Regular Migration; [3]). Objective analysis is especially crucial in the light of non-peer-reviewed and readily accessible alternative documents (e.g., [31–33]), often by organizations promoting a particular point of view or ideology, that gain credibility when integrated into the primary literature (e.g., [16]).

Achieving UNFCCC targets will not be easy for any nation, and perhaps especially so for Canada and the USA where high emissions are variously linked to high consumer demand, energy production and the constraints of geography, climate, and expanding populations. Indeed, the cumulative reduction in greenhouse gas (GHG) emissions by Paris Agreement Parties is expected to fall far short of COP21’s goal to limit global temperature rise to 1.5°C (GHG emissions only 0.7% lower than in 1990; [34]). The evidence thus suggests that Canada and the USA are more likely to achieve their original pledges than their 2021 announcements.

Although all greenhouse gases and their sources are included in pledges, the most obvious target is to reduce CO_2_ emissions associated with the production, transportation and combustion of fossil fuels. Fossil fuel consumption is related directly or indirectly to per capita demand, and thus to sources of population change, including migration. Accordingly, I seek to evaluate the potential of migration-driven population dynamics on nations’ abilities to meet their original 2030 emission targets. The predictions can help inform policies that enable nations to achieve ever more ambitious climate-change objectives and migration expectations.

### Estimating effects of migration on CO_2_ emissions

The challenge is to develop predictive metrics that merge human migration with nations’ plans to limit CO_2_ emissions. Ideally, one would like to meet the challenge by first identifying and quantifying the full list of causal mechanisms that link migration’s effects on human demography with economic, political and social strategies (the statistical model in [18] is a good start). Doing so would enable understanding of their connections with population growth and their relationships with CO_2_ emissions. One could then use the mechanisms to forecast national as well as global future scenarios on CO_2_ reduction. But migration is also influenced by a variety of unpredictable events including internal and external conflicts, natural disasters, and a host of unplanned economic and political exigencies. These difficulties limit our ability to build better models that nevertheless require time we can ill afford to waste while global demand for energy and its manifold effects on climate and other Earth systems continue to rise. I offer a more immediate alternative that uses publicly available data to forecast likely consequences of human migration on nations’ ability to meet their respective UNFCCC 2030 targets. I do so in four distinct ways.

1. Assess the functional relationship between countries’ CO_2_ emissions and human population size.
2. Reveal the most likely determinants of global human migration with special emphasis on whether migrants tend to move from low-CO_2_-emitting to higher-emitting countries. If they do, and if analyses confirm the assumption that CO_2_ emissions increase with increasing population size, then it is reasonable to assume that international human migration yields a net increase in global emissions.
3. Assess the net effect by predicting global CO_2_ emissions in a World with and without migration. The predictions assume the best-case scenario that, beginning in 2021, all nations reduce their 2005 CO_2_ emission levels by 3% annually (= 30% reduction in 2030 in general agreement with the UNFCCC [1]). The assumption guarantees that any effect attributed to migration will not overestimate its true impact. I also assume that all population growth (positive for net immigrating countries, negative for net emigrating countries) until 2030 can be attributed to migration. This assumption guarantees that per capita calculations related to migration include only the effect of migration uncompromised by internal population dynamics.
4. Assess the expected impact of immigration on cumulative CO_2_ emissions for Canada and the USA under two scenarios: 1, that, beginning in 2021, each nation achieves a 3% reduction in emissions annually in order to meet the 30% reduction by 2030 and 2, that neither nation is successful in reducing emissions. A comparison of the two scenarios helps to calibrate the likely impacts of immigration relative to the ability of each country to reduce its reliance on fossil fuels.

I conclude by considering the consequences of each projection on meeting UNFCCC climate targets.

## Materials and methods

I obtained data for both global and Canada/USA analyses from readily available national and international data sources (e.g., United Nations, World Bank) and repositories (S1 and S2 Tables). The assumptions involved in collecting the data, and the potential for errors, are described fully in the respective web pages and source documents.

I use 2021 as the starting point for projected impacts of immigration because final statistics for 2020 await analysis and release from their appropriate government sources, and because the 2020–2021 COVID-19 pandemic reduced international migration (and temporarily reduced CO_2_ emissions [35]). The prognostications make numerous, but reasonable, assumptions regarding temporal patterns of migration and population growth, equivalent energy use between immigrants and residents, and the dependence of CO_2_ emissions on population size (revisited in the discussion). I outline general methods below. Details of each method and its assumptions are fully described in S1 Appendix.

### Global analysis

I began by assessing the functional relationship between a country’s CO_2_ emissions and its population size. I did so by applying linear, quadratic and cubic regressions of CO_2_ emissions against human population size for the period 1960–2014 (201 countries; 603 regressions; includes Hong Kong and Macao). I repeated the analysis on the global total and interpreted whether the significance and general shapes of each regression represented increasing or decreasing trends in emissions with population size.

I then accessed the most recent UN data [17] on human migration (2015–2020) to determine the ‘current’ rate of emigration. I downloaded international migration statistics, and a sub-set of predictor variables representing climate change, economic, and demographic statistics, from Our World in Data’s SDG-Tracker website [36; Table 1]. Climate change impacts virtually all life on Earth, so I included an additional metric representing each nation’s attempts to conserve biodiversity. The assumption in doing so is that nations conserving biodiversity might represent attractive targets for immigrants. I used stepwise logistic regression to identify which of these global predictors of human migration are associated directly, or indirectly, with CO_2_ emissions.

**Table 1:** List of national binary variables used to assess general patterns of human global migration.

Variable	Type and Coding
Net Migration between 2012 and 2017	Dependent: 0 < 0 (104 nations), 1 ≥ 0 (71 nations).
Per Capita GDP (constant 2011 international $, WorldBank, 2014)	Predictor: 0 = less than median, 1 ≥ median
CO_2_ Emissions (tonnes per capita in 2014)	Predictor: 0 = less than median, 1 ≥ median
Per Cent of Key Biodiversity Areas in Protected Areas (2014)	Predictor: 0 = less than median, 1 ≥ median
Population Growth Rate (%) without Migration	Predictor: 0 = less than median, 1 ≥ median
Population Density in 2014 (people·km^-2^)	Predictor: 0 = less than median, 1 ≥ median
Projected Population Growth (%) in 2050	Predictor: 0 = less than median, 1 ≥ median
Population Size (2014)	Predictor: 0 = less than median, 1 ≥ median
Projected Population Size in 2100	Predictor: 0 = less than median, 1 ≥ median

All data downloaded from SDG-Tracker.org. Full descriptions available at https://ourworldindata.org.

I used the sums of CO_2_ emissions for net emigrating and net immigrating nations to calculate the expected global difference in CO_2_ emissions associated with emigration. I used these values to calculate the expected impact on attaining CO_2_ reduction targets by 2030.

### Analyses for Canada and the USA

I explored the expected impact of migration on Canada and the USA by using a combination of publicly available UN, WorldBank, and national statistical data. I compiled the number of annual immigrants to Canada [37], and separately to the USA [38], based on their country of birth (213 and 207 states and territorial entities respectively). I used these data to cumulate the anticipated effect of immigration on attaining Canadian and USA CO_2_ emission targets between 2021 and 2030. I cumulated separate values for the best-case scenario in which all nations achieved a 30% reduction in CO_2_ emissions relative to 2005 levels, as well as for the zero reduction alternative. All other reasonable options to reduce emissions lie within these limits. CO_2_ emissions are a global issue, so I also calculated the weighted per cent of global energy use of potential emigrants under the assumptions that they do not migrate to Canada or to the USA.

These simple accountings, as well as those for the global estimates, include real and potential sources of error that are explained fully in S1 Appendix. I assume that data and accounting inaccuracies yield no systematic bias that compromises conclusions on the relative effects of immigration on CO_2_ emissions.

## Results

### CO_2_ emissions increase with population size

The vast majority of countries (148 of 201) had a full compliment of data for the entire period between 1960 and 2014. Twenty-eight countries, dominated by those ‘liberated’ by the breakup of the Soviet Union in 1991, had less than 25 years of data.

**Fig 1:**
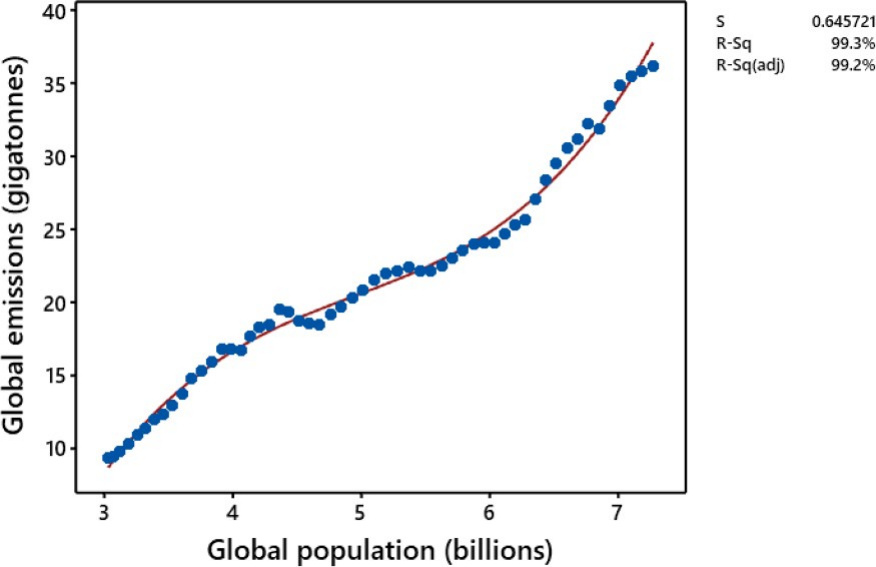
The cubic relationship between global CO_2_ emissions and world human population size (1960–2014; *Y* = 59.92 *X–* 11.4 *X*^2^ + 0.77 *X*^3^–89.27; *R*2adj = 0.992; *N* = 55). Line = fitted curve, dots = data.

Most relationships between CO_2_ emissions and population size yielded excellent fits to the data (mean *R*^*2*^ adjusted for sample size = 0.81) and most (120) were best fitted with cubic models. Only two nations, both with relatively short sequences of data, yielded non-significant relationships (Germany, 23 yr; Montenegro, 10 yr). Statistically significant linear terms were predominantly positive (167 countries). Despite vagaries in CO_2_ emissions through time, CO_2_ emissions accelerated with human population size in 129 countries (convex [+ve squared term in a quadratic model) = 31 countries; sigmoidal upward [+ve cubed term in a cubic model] = 88 countries), a pattern that is also reflected in the cumulated global data (Fig 1). CO_2_ emissions during the past half century increased with expanding human populations in most countries, in the world as a whole, and often at an accelerating rate.

The relationship between CO_2_ emissions and population size in Canada was also sigmoidal upward with a positive cubed term, but that in the USA was best fit by a quadratic model with a negative squared term (S2 Appendix). The apparent levelling off in the USA is driven primarily by a 5-year reduction in emissions following the 2008 economic downturn. Despite the differences between the two nations, the most recent data (2012–2014) document an increase in emissions in both countries. Even so, we can expect another dip in emissions caused by reduced economic activity associated with COVID-19. Those effects may reduce the baseline CO_2_ emissions per capita (similar to reaching UNFCCC climate targets that I model here), but not the reality that, for any baseline, more people require more energy.

### Global trends

The global number of international migrants in 2019 (271.6 million) reflects a continuing trend of ever increasing human migration [17]. The United States has more migrants (50.7 million) than any other country, but the proportion of the total population composed of migrants (0.154) is substantially less than Canada’s 0.213 (with 8 million migrants; [17]). Both proportions far exceed the global mean of 0.035 [39]. Global net emigration (sum of the net outflow of humans from 104 emigrant countries; [17]) between 2012 and 2017 totalled 16.71 million people (~ 3.34 million annually; the actual number of migrants is much higher).

On a global scale, the likelihood of a nation being a net exporter of migrants in 2014 was about ten times higher in nations with high population growth rates in the absence of migration than in others (logistic regression, *N* = 175; response event = 0 [net emigration]; odds ratio = 9.77; *p* = 0.037). It was much lower in nations with relatively high per capita GDP and projected high rates of growth in 2050 (odds ratios = 0.034; *p* < 0.001 and 0.046; *p* = 0.006 respectively; Table 2). The opposite holds true for countries with net immigration.

**Table 2:** Statistically significant predictors (defined in Table 1) of migration status.

Source	Degrees of Freedom	Chi-square	*p*	Odds Ratio
Analysis Including Binary GDP Per Capita (Deviance R-Sq_adj_ = 33.74%)				
Regression	3	82.75	< 0.001	
Per Capita GDP	1	45.24	< 0.001	0.0384
Population Growth Rate without Migration	1	6.60	0.01	9.7732
Projected Population Growth in 2050	1	14.84	< 0.001	0.0465
Error	171			
Analysis Excluding Binary GDP Per Capita (Deviance R-Sq_adj_ = 22.84%)				
Regression	1	54.98	< 0.001	
CO_2_ Emissions	1	54.98	< 0.001	0.0786
Error	173			

Binary logistic regression: 0 = net emigration; 1 = net immigration (or balance between emigration and immigration; Angola, Azerbaijan, Bhutan, Jordan, Kazakhstan, Mauritius); 175 nations.

**Fig 2:**
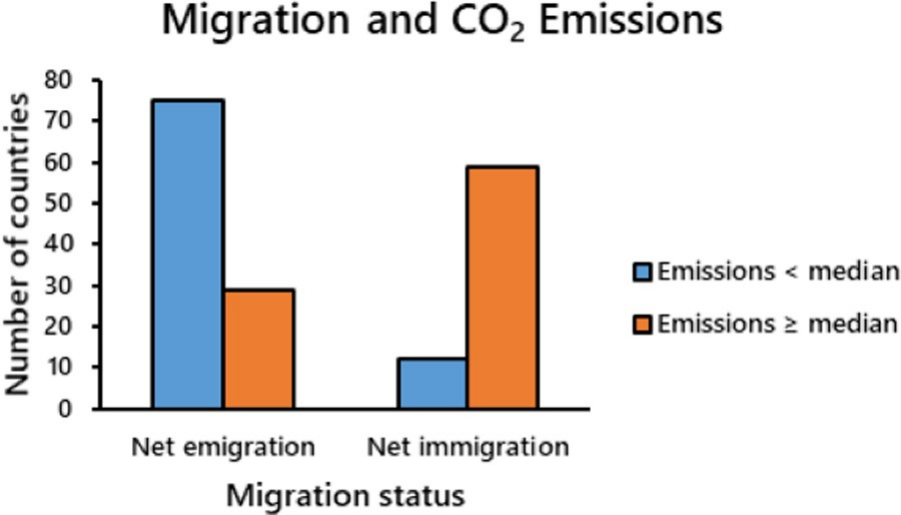
Frequency of countries with below and above median per capita CO_2_ emissions in 2014 that were classified with either net human emigration or net immigration during the 5-yr interval between 2012 and 2017 (*N =* 175; data from https://ourworldindata.org; downloaded 15 December 2019).

The patterns changed dramatically when I removed the redundant binary indicator for per capita GDP (S1 Appendix) from the analysis. The likelihood of a nation being a net exporter of human migrants was close to 13 times greater (odds ratio = 0.079; *p* < 0.001) in nations with low per capita CO_2_ emissions than in nations with high emissions (Table 2 and Fig 2).

**Fig 3:**
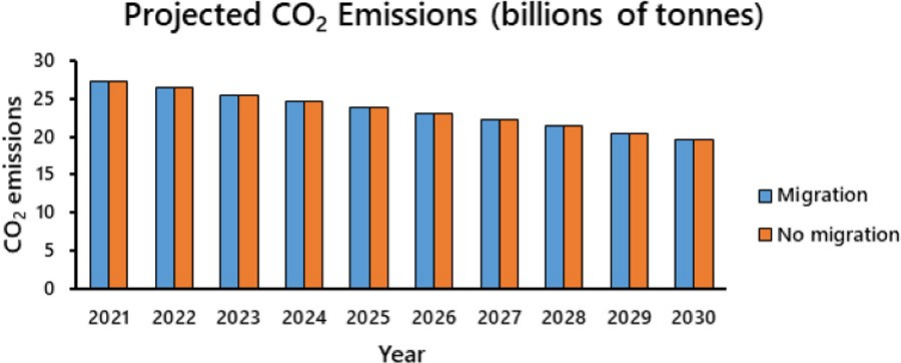
Projected CO_2_ emissions attributable to global human migration from 2021 to 2030 (assumes growth only through migration and that all nations achieve a 30% reduction relative to 2005 levels; *N =* 175; values calculated with data obtained from https://ourworldindata.org; downloaded 15 December 2019).

Total CO_2_ emissions of 104 ‘emigrant’ versus 71 ‘immigrant’ countries were similar in 2014 (approximately 16.6 vs 17.3 gigatonnes [gt]). But differences in population size created a weighted mean per capita that was nearly three times larger (8.84 tonnes) in immigrant countries than in emigrant nations (3.17 tonnes). Assuming that all nations reduce emissions to 70% of 2005 levels, the projected cumulative increase in net emissions caused by first-year immigrants (~158 megatonnes [mt]) corresponds to an approximate increase of 0.07% over that which would occur in the absence of migration (234.2 gt; Fig 3). The decadal cumulative effect, accounting for future net emissions by immigrants, is much larger, (~ 1.7 gt) but it is still a small proportion (~ 0.7%) of the 234 gt total without migration. Although the effect of migration in reducing CO_2_ emissions pales in comparison to what is possible via the Paris agreement, all reductions have merit in combating climate change.

### Canada and the USA

In 2014, Canada (15.16 tonnes) and the USA (16.5 tonnes) ranked among the world’s highest per capita emitters of CO_2_ (13 and 9 respectively). In terms of total emissions, China (10.3 gt CO_2_), emitted nearly twice that of the second largest emitting nation (United States, 5.3 gt). Canadian emissions were about 1/10^th^ those of the USA (537 mt; global rank = 12). Together, Canada and the USA emitted approximately 16% of the global total of 36.1 gt CO_2_.

The 2014 net immigration rate was substantially higher in Canada (0.65%) than in the USA (0.28%). U.S. immigrants tended to come from slightly less energy intense countries than did those entering Canada (weighted mean tonnes CO_2_ per capita of immigrants entering the U.S. = 3.72 versus 4.12 tonnes in Canada). This difference was maintained even though more Canadians emigrated to the USA (11,484) than did USA citizens immigrating into Canada (8,491).

**Fig 4:**
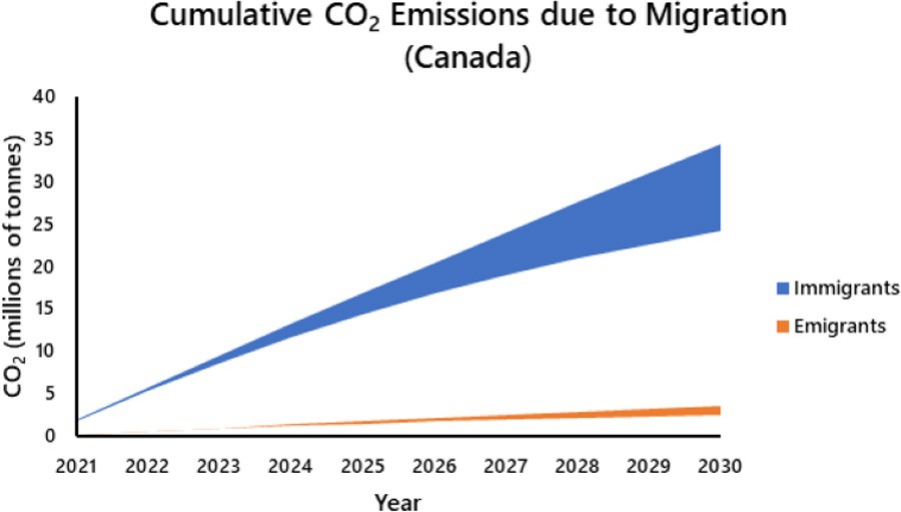
Projected annual cumulative CO_2_ emissions associated with migration to and from Canada for the period 2021 to 2030. The upper limit of each curve assumes that Canada maintains 2005 emission levels, the lower limit assumes that Canada achieves an annual reduction of 3% (= 30% reduction by 2030). Shading represents the range of possibilities for partial achievement of reduction targets.

**Fig 5:**
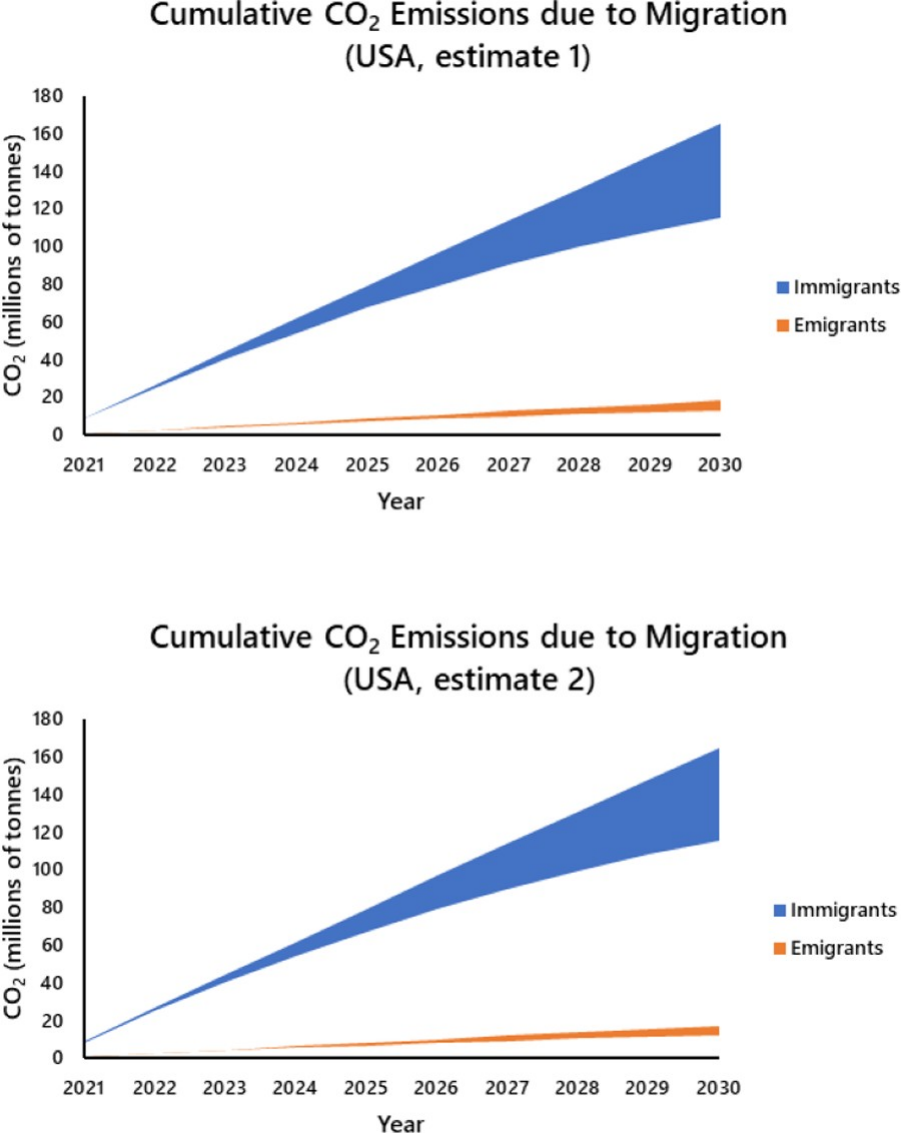
Projected annual cumulative CO_2_ emissions associated with migration to and from the USA for the period 2021 to 2030. The upper limit of each curve assumes that the USA maintains 2005 emission levels, the lower limit assumes an annual reduction of 3% (= 30% reduction by 2030). Shading represents the range of possibilities for partial achievement of reduction targets. S1 Appendix provides a detailed explanation of the calculations.

**Fig 6:**
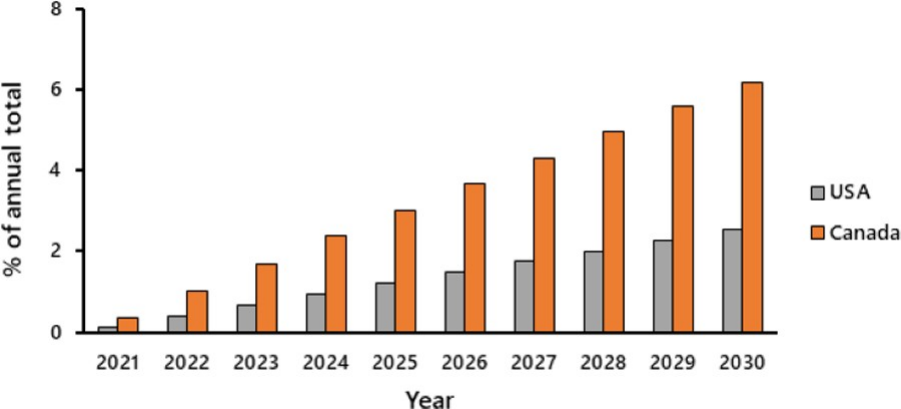
Projected annual percent contribution of net immigration to annual CO_2_ emissions in Canada and the USA from 2021 to 2030 (uses USA emigration estimate 1 and assumes that both Canada and the USA attain an annual 3% reduction in 2005 emission levels [= 30% reduction by 2030]).

The annual projected difference in CO_2_ emissions associated with immigration versus emigration declined annually in both countries, but the multiplicative effect over time yielded an ever expanding difference in cumulative CO_2_ emissions associated with immigrants versus emigrants (Figs 4 and 5). The projected total emissions from 2021 to 2030 attributable to international migration alone is approximately 745 mt (Canada = 130 mt [rises to 165 mt with no CO_2_ reduction; the values increase dramatically to ~ 175 and 225 mt (S2 Appendix) if future Canadian immigration equals the 341,000 total received in 2019 [40]; USA using emigration estimate 1 = 613 mt; estimate 2 = 617 mt [rise to 779 and 784 mt respectively with no CO_2_ reduction; estimates explained fully in S1 Appendix]). Even at 2014 immigration levels, the cumulative contribution equates with approximately 33% of Canada’s projected 2030 UNFCCC emissions (390 mt) in the absence of migration and growth, and approximately 15% of that projected for the USA (4.053 gt). On an annual basis, the multiplicative effect of immigration also represents a non-trivial component of projected CO_2_ emissions by Canada and the USA (Canada: rises to 6.18% of annual emissions by 2030; USA: rises to 2.54%, Fig 6).

Global percentages are, of course, much smaller. The annual effect of immigration to Canada and the USA reaches a maximum of approximately 1% of global emissions by 2030 under the ‘no change’ scenario (S2 Appendix). It is somewhat smaller (0.7%) if all nations achieve 30% reduction targets (S2 Appendix). In order to appreciate the total impact, we must compare these values against those that would occur in the absence of immigration. These percentages are lower, totalling approximately 0.8% of global 2030 emissions (no change) and 0.7% (meet 30% reduction targets) respectively. The net effect of immigration to Canada and the USA is thus between 0.1% and 0.2% of expected global emissions in 2030.

## Discussion

Human populations, as in all others, change size through births, deaths and migration. Humans migrate among and within countries for myriad reasons that nevertheless reflect adaptive responses to their density-dependent fitness prospects [26,41]. It is thus hardly surprising that human migration is increasing in a world with ever-growing human populations exhibiting wildly different social, economic and evolutionary potential.

Ignoring all other perspectives, the role of human numbers has its least effect on climate change in nations with low CO_2_ emissions. Paradoxically, emigrants from those nations are small but significant additional contributors to the global consumption of fossil fuels and its associated atmospheric pollution. Those contributions mirror theory [42] and data [26,43] demonstrating the overarching importance of density and energy as determinants of human migration, distribution and globalization.

Increased human population size not only correlates strongly with their nations’ CO_2_ emissions, those emissions have typically increased more rapidly than expanding population size (also noted by Shi [44]). It is thus reasonable to assume that increases in population size, whether by migration or internal dynamics, increase CO_2_ emissions in the short term. It follows that human migration can contribute to the difficulties in meeting global CO_2_ reduction targets by 2030, and especially so in nations with currently high per capita emissions.

Longer term effects are more difficult to forecast because increased global warming is likely to reduce economic activity [45,46] and thus, CO_2_ emissions. These predictions are complicated by indirect effects and the effects of other determinants of CO_2_ emissions [47,48]. Indeed, climate-induced reductions in per-capita productivity are expected to have a far greater effect on reducing CO_2_ emissions by 2100 than do co-determinants of population size, energy intensity, or the carbon intensity of the energy that nations use [48]. Although these, other factors (including pro-active policies to reduce emissions [48]), and temporal variation complicate long-term forecasts, they are much less likely to impinge on the population-dependent ability of nations to achieve CO_2_ reductions by the 2030 timeframe used here.

The forecasts carry two additional and related caveats. 1. That ‘average’ per capita effects represent the expected contributions to CO_2_ emissions by both migrants and residents. 2. That decisions on migration, and behavior following immigration, reflect the norms of the respective emigrant and immigrant nations. If the majority of emigrants represent high CO_2_ ‘emitters’ in their home countries, then emigration might further reduce net national emissions. On the other hand, if immigrants tend to ‘emit’ less CO_2_ than residents, then the forecasts overestimate the net effect of immigration. Incorporating such effects will be difficult to impossible because they invoke a wide spectrum of interacting social, political, economic and individual factors. Regardless, current evidence is that immigration tends to increase per capita GDP (S1 Appendix) and its knock-on effects of CO_2_ emissions. The use of population averages is thus more likely to underestimate, than overestimate, immigrations’ influence on meeting climate-change objectives.

Although calculated totals due to migration are measured in millions (Canada and the USA) to as much as 1.7 billion tonnes on a global basis, they represent relatively small proportions of current and projected total emissions. One can thus argue that, relative to the magnitude of the overall problem, and the relatively low contribution anticipated for population size in future [48], there is no need to address the effects of migrants on CO_2_ emissions. The influence of human migration is trivial in comparison with efforts to limit climate change through global reductions in fossil fuels, and feedbacks onto economic productivity. While the comparison is undoubtedly true, so too is the fact that societies and countries must use every mechanism available to them if they are to increase energy efficiency, reduce demand, and minimize the negative consequences of difficult social, economic, and political decisions. This is especially true in high CO_2_ emitting nations, such as Canada and the USA, where every additional individual, whether via birth or immigration, has the greatest average per capita influence [12,14]. Policies to meet CO_2_ emission targets must include the anticipated contribution associated with migrating humans. An example of this importance can be found in Canada’s much heralded decadal plan to plant two billion trees with a projected annual reduction in greenhouse gas emissions of 12 mt by 2050 [49]. Cumulated over the subsequent decade, the sum is on the same order as the 2021–2030 total for the effects of immigration calculated here. Policies designed to meet migration targets must recognize the interconnection with policies on climate change and that changes to either policy have significant implications for the other.

No matter what opinions and policies may emerge from these analyses, there can be no doubt that, under current conditions, more people equates with more demand on energy. In this context, a few hundred million human migrants contribute far less to CO_2_ emissions than does population growth (e.g., UN’s median projections yield 8.5 billion humans in 2030, 9.7 billion in 2050, and 10.9 billion in 2100; maximum estimates are much higher [50]; forecasts by Vollset et al. [18] are much smaller). That said, we must also recognize that proximate determinants of human migration reflect causes rooted deeply in population density [26,41].

Ceteris paribus, energy demand and CO_2_ emissions depend on population size. Even if human migration is a minor inhibitor to attaining UNFCCC targets, it is a symptom of the much greater problem of living during a time when too many people consume resources with negative feedbacks on the Earth System [51–54]. It is also a problem made more difficult by disparities in the distributions of resources, energy use, wealth, opportunity, and commitment. CO_2_ emissions are disproportionate to the wealth of nations, and the relatively minor problems faced by immigrant countries such as Canada and the USA in attaining UNFCCC targets are unlikely to gain much sympathy from poorer less consumptive countries compromised by climate change and high human densities. One must ponder whether it is legitimate to ask poorer countries to control emissions unless rich countries first demonstrate a concerted commitment to reduce consumption.

CO_2_-related human migration is likely to increase as climate-induced environmental degradation adds to the global challenges of human movement (e.g., [55]). Attributing cause and effect is misleading in the sense that connections between climate change and migration emerge via a nexus of intervening factors (e.g., [56]) that influence both internal and international migration through variation in human mobility [29]. The proportion of internal migrants, that greatly exceeds the proportion of people moving among countries, is likely to increase with the hardening of international boundaries designed to choke off immigration [57]. Even so, worsening climate and continued population growth will exacerbate the difficulties faced not only by migrants, but also by countries trying to invoke the Global Compacts. Reductions in CO_2_ emissions in high-emitting nations will mitigate those difficulties only if they merge with policies aimed at improving the wealth and adaptive capacities of poor nations [30,57]. Solutions, as with other significant environmental challenges associated with human population dynamics, require that nations place cooperative actions above national interest [54]. Those actions must simultaneously seek to reduce CO_2_ emissions, address inequities in consumption, provide improved and clear opportunities for the world’s poor, and turn back the tide of increasing human population size.

## Supporting information

S1 Table Provides details, source and access dates of general data used in this contribution. (DOCX)

S2 Table Provides details, source and access dates for data used in the logistic regression analyses. (DOCX)

S1 Appendix Provides a detailed description of methods and assumptions. (DOCX)

S2 Appendix Provides additional illustrations of the impact of migration on CO_2_ emissions. (DOCX)
