# Supplementary material for: On the effect of international human migration on nations’ abilities to attain CO2 emission-reduction targets
Source: PLoS One. 2021 Oct 4;16(10):e0258087. doi: 10.1371/journal.pone.0258087 (PMC8489703; doi:10.1371/journal.pone.0258087)
Supplement: S1 Table — (DOCX) [file pone.0258087.s001.docx]

**Table S1.** Descriptions and sources of general data used in “The effect of human migration on attaining CO_2_ emission-reduction targets”.

| **Data description** | **Source** | **Access date** |
| --- | --- | --- |
| Canada Emigration 2014/2015 | Statistics Canada. Table 17100008. <https://www150.statcan.gc/n1/tbl/scv/17100008-eng.zip> | 5/12/2019 |
| Canada Immigration Statistics | Research and Evaluation Branch, Immigration, Refugees and Citizenship Canada. Table IRCC_FF_PR_2017_15. <https://open.canada.ca/data/en/dataset/082f05ba-e333-4132-ba42-72828d95200b> | 28/11/2019 |
| Canada Population Size 1 July 2019 | Statistics Canada. Canada’s population estimates: age and sex, July 1, 2019. <https://www150.statcan.gc.ca/n1/en/daily-quotidien/190930/dq190930a-eng.pdf?st=BzQrnfIl> | 2/12/2019 |
| CO_2_ Emissions Per Capita | World Development Indicators, The World Bank. Table 68. [https://databank.worldbank.org/reports.aspx?source=2&series=EG.USE.PCAP.KG.OE&country=#](https://databank.worldbank.org/reports.aspx?source=2&series=EG.USE.PCAP.KG.OE&country=) | 27/11/2019 |
| Energy Production (Total and Per Capita) | Global Change Data Lab. Our World in Data. University of Oxford. Files: fossil-fuel-production-over-the-long-term.csv; fossil-fuel-production-per-capita.csv; <https://ourworldindata.org/fossil-fuels#fossil-fuel-production> | 13/12/2019 |
| Gasoline equivalent | Natural Resources Canada. Learn the facts: fuel consumption and CO_2_. <https://www.nrcan.gc.ca/sites/www.nrcan.gc.ca/files/oee/pdf/transportation/fuel-efficient-technologies/autosmart_factsheet_6_e.pdf> | 12/12/2019 |
| Global migration | Migration data portal. <https://www.migrationdataportal.com/data?i=netmigrate&t=2020>; source data from the United Nations Department of Economic and Social Affairs (UN DESA) | 12/12/2019 |
| Population Sizes | World Development Indicators, The World Bank. [https://databank.worldbank.org/indicator/SP.POP.TOTL/1ff4a498/Popular-Indicators#](https://databank.worldbank.org/indicator/SP.POP.TOTL/1ff4a498/Popular-Indicators) | 3/12/2019 |
| Total CO_2_ emissions | World Development Indicators, The World Bank. <https://data.worldbank.org/indicator/EN.ATM.CO2E.KT> | 3/12/2019 |

**Table S1 continued.** Descriptions and sources of general data used in “The effect of human migration on attaining CO_2_ emission-reduction targets”.

| **Data description** | **Source** | **Access date** |
| --- | --- | --- |
| Total CO_2_ emissions Canada and USA 2015 and 2017 Calculations | Global Change Data Lab. Our World in Data. University of Oxford. File: annual-co2-emissions-per-country.csv; <https://ourworldindata.org>;  Hannah Ritchie and Max Roser (2019) - "CO₂ and Greenhouse Gas Emissions". Published online at OurWorldInData.org. Retrieved from: 'https://ourworldindata.org/co2-and-other-greenhouse-gas-emissions' | 31/12/2019 |
| USA immigration statistics | U.S. Department of Homeland Security. Table fy2017_table3d. <https://www.dhs.gov/immigration-statistics/yearbook/2017> | 27/11/2019 |
| USA population size 1 July 2019 | United States Census Bureau. U.S. and World Population Clock. <https://www.census.gov/popclock/> | 5/12/2019 |
