# Supplementary material for: On the effect of international human migration on nations’ abilities to attain CO2 emission-reduction targets
Source: PLoS One. 2021 Oct 4;16(10):e0258087. doi: 10.1371/journal.pone.0258087 (PMC8489703; doi:10.1371/journal.pone.0258087)
Supplement: S2 Table — (DOCX) [file pone.0258087.s002.docx]

**Table S2.** Descriptions and sources of data used in logistic regression analyses of migration in “The effect of human migration on attaining CO_2_ emission-reduction targets”.

| **Data description** | **Source** | **Access date** |
| --- | --- | --- |
| Net migration | Global Change Data Lab. Our World in Data. University of Oxford. File: net-migration.csv; <https://ourworldindata.org> | 15/12/2019 |
| Per capita CO_2_ | Global Change Data Lab. Our World in Data. University of Oxford. File: co-emissions-per-capita.csv; <https://ourworldindata.org> | 15/12/20190 |
| Per capita GDP | Global Change Data Lab. Our World in Data. University of Oxford. File: gdp-per-capita-worldbank.csv; <https://ourworldindata.org> | 15/12/2019 |
| Population density | Global Change Data Lab. Our World in Data. University of Oxford. File: population-density.csv; <https://ourworldindata.org> | 15/12/2019 |
| Population growth | Global Change Data Lab. Our World in Data. University of Oxford. File: population-growth-rates.csv; <https://ourworldindata.org>  Max Roser, Hannah Ritchie and Esteban Ortiz-Ospina (2019) - "World Population Growth". Published online at OurWorldInData.org. Retrieved from: 'https://ourworldindata.org/world-population-growth' | 16/12/2019 |
| Population size | Global Change Data Lab. Our World in Data. University of Oxford. File: projected-population-by-country.csv; <https://ourworldindata.org> | 16/12/2019 |
| Protected terrestrial biodiversity areas | Global Change Data Lab. Our World in Data. University of Oxford. File: protected-terrestrial-biodiversity-sites.csv; <https://ourworldindata.org> | 15/12/2019 |
