# Supplementary material for: On the effect of international human migration on nations’ abilities to attain CO2 emission-reduction targets
Source: PLoS One. 2021 Oct 4;16(10):e0258087. doi: 10.1371/journal.pone.0258087 (PMC8489703; doi:10.1371/journal.pone.0258087)
Supplement: S1 Appendix — (DOCX) [file pone.0258087.s003.docx]

**S1 Appendix: Detailed Methods and Assumptions**

*CO_2_ emissions and human population size*

I calculated linear, quadratic and cubic regressions of CO_2_ emissions versus population sizes of 201 countries (includes Hong Kong and Macao; MINITAB 19). I repeated the analysis on the cumulated global data. I chose the ‘best’ model as the one with the highest significant polynomial term; if neither quadratic nor cubic fits were statistically significant, I evaluated only the linear term. This procedure retained some models in which a lower term (e.g., quadratic or linear effects in a significant cubic model) were not statistically significant. I did so because I aimed to use the “best-fitted” model to quantify and visualize the most probable recent trends in the relationship between emissions and population size. Regardless, I also evaluated all linear fits in order to determine general trends of increasing or decreasing dependence of emissions on population size.

*Global trends*

I used Our World in Data’s SDG-Tracker website’s (Our World in Data 2019) most recent data on migration (five years previous to 2017), but when appropriate, analyzed predictor variables from 2014 because they corresponded with the most recent data available from other sources (below).

Frequency distributions of the data were often highly skewed or otherwise non-normal, so I converted all potential predictors into binary variables coded as 0 for values less than the median and 1 otherwise. I created a similar dependent variable coded 0 for countries with net emigration versus 1 for the remainder. I then searched for general predictors of human migration with stepwise logistic regression (MINITAB 18; *α* to enter = 0.05; *α* to remove = 0.1). My goal was to identify reliable global predictors of human migration, not to build the overall ‘best’ statistical model of human migration, or to identify and prioritize the many social, cultural, economic, geopolitical and regional drivers of migration.

The stepwise solution did not include the anticipated direct effect of CO_2_ emissions on human migration. I reasoned that this might reflect a high correlation between per capita GDP and per capita CO_2_ emissions (*r =* 0.83; *p* < 0.001; even higher on log-transformed data; *r =* 0.91). So I repeated the analysis after deleting the binary variable representing per capita GDP. My intent was to assess whether CO_2_ emissions depend on human migration, not to downplay the interconnection of both variables with per capita GDP.

I estimated cumulative global effects of human migration on CO_2_ emissions until 2030 by calculating separate sums of the total 2014 CO_2_ emissions for nations with net emigration, versus those with net immigration. I imagined that changes in a nation’s human population size was caused only by migration in order to isolate its sole impact on CO_2_ emissions. I also assumed that the annual number of emigrants matched the mean annual emigration between 2012 and 2017 (UN DESA 2019a).

My interest was not simply to calculate annual rates of CO_2_ emissions, but was instead to determine the expected impact on achieving CO_2_ reduction targets in 2030. I was concerned the annualized data, that imagine all migrants depart at the end of the year, would induce time lags in my calculations. I eliminated this effect by dividing the expected number of annual emigrants by two. This calculation assumes that the average migrant spends one-half of the year in the home country, and the remaining half in the new country (this calculation is equivalent to a constant rate of migration throughout the year; e.g., some migrants spend 1 month ‘at home’, others 11 months — the mean across all migrants is 6 months). I did the same for receiving countries (divided net immigrants by two). I subtracted the residual one-half of emigrants from emigrating countries from, and added the same number as immigrants to immigrating countries to, the subsequent year’s starting populations (implies that they migrated during the remainder of the current year) before iteratively recalculating the mid-year value for the subsequent year. I then calculated the expected global difference in CO_2_ emissions associated with emigration relative to that without for each year, as well as decadal totals. These calculations are likely highly conservative of true effects for at least two reasons. 1, the number and proportion of human migrants have increased dramatically in the past decade (UN DESA 2019b), as has human population size (UN DESA 2019c) and 2, markedly higher energy use in richer nations receiving immigrants provides more opportunity for total reductions in CO_2_ emissions (increased likelihood to reach 2030 targets) than in poorer nations with net emigration.

*Analyses for Canada and the USA*

I used the sum of all immigrants from the most recent available data (2014) as a measure of ‘current’ immigration (Canada = 259,833; USA = 1,016,160). I assumed that the Canada and USA populations between 2020 and 2030 increased only through immigration and that per capita CO_2_ emissions by immigrants equaled those of ‘residents’. The first assumption enables an estimate of the effect of immigration alone. The second seems reasonable given that the baseline emissions statistics I use include the impact of recent immigrant Canadians and Americans who represent significant proportions of each country’s population (21% in Canada; 15% in the USA; UN, DESA, 2019a; Statistics Canada reported a somewhat higher proportion [23.9 % in 2016, Statistics Canada, 2017]).

Recent immigrants tend to earn less than residents in both Canada (Statistics Canada 2018) and in the USA (Radford and Noe-Bustamante 2019), an effect that might reduce their CO_2_ footprint. But immigrants also tend to represent a greater proportion of working-age adults than residents (Jaumotte et al. 2016), and contribute disproportionately to entrepreneurial activities (Green et al. 2016). These and other effects act to increase per capita GDP (Jaumotte et al. 2016). The economic impacts of immigrants, if anything, suggest that the assumption of equal per capita contributions to CO_2_ emissions might be too conservative.

As in the global analysis, I divided the number of immigrants arriving in a given year by two on the assumption that the ‘average’ arriving immigrant spends six months at ‘home’ and six months in Canada or the USA. I multiplied this number by the per capita CO_2_ emission (respectively for Canada and the USA) for that year in order to determine the total emissions associated with newly arriving immigrants. As in the global projections, I added the remaining one-half of immigrants to the yearly total before iterating calculations for the subsequent year. The data do not include demographic details, or estimates of return migration (but return migration is a minor component of the net flow of migrants into Canada and the USA [e.g., McAuliffe et al. 2020] and is included indirectly in estimates of annual emigration out of Canada and the USA). I thus assumed that beginning in the year after their immigration, each cohort of immigrants maintained its population size (births perfectly compensate for morality). I then multiplied that number times Canadian and USA estimates of per capita CO_2_ emissions. I subtracted the number of emigrants leaving Canada and USA multiplied by their respective per capita emissions, to yield the expected CO_2_ emissions associated only with net immigration in that year.

I summed these values across years to yield the total expected impact of immigration on CO_2_ emissions during the interval 2020 to 2030. My calculations excluded former emigrants who maintained citizenship and returned to Canada and the USA. This simple accounting, as well as that for the global estimates, includes numerous real and potential sources of error. I assume that data and accounting inaccuracies yield no systematic bias that compromises conclusions on the relative effects of immigration on CO_2_ emissions.

I was unable to find publicly available first-hand data on USA emigration, and no data that I could use to estimate 2014 emigration directly. So I used the U.S. Department of Homeland Security data on lawful permanent residents entering the U.S. in 2017 (1,127,167), subtracted the estimated net international migration that year estimated by the U.S. Census Bureau (Projected population size and births, death, and migration, Projections for the United States: 2017-2060, Main Series. Table 1. [np2017-t1: 1002,000]) to yield the expected emigration of 125,167 U.S. residents abroad. I converted this value to a proportion of the total permanent residents entering the U.S. that year (125,167/1,127,167 = 0.111046). I then applied the same percentage to the 2014 immigration value of 1,016,160 = 112,840 (emigration estimate 1).

I undertook another estimate in which I assumed that the USA emigration rate (relative to the number of immigrants) was the same as that estimated for Canada. I did so because the two countries possess inter-dependent economies, they have common histories of human migration, and similar values of freedom and democracy. This approach yielded a comparable value of 107,074 emigrants (estimate 2). I used both estimates in separate calculations. Both estimates should yield conservative estimates of immigrant contributions to CO_2_ emissions because they do not include illegal entrants.

In late 2019, the mandate letter to Canada’s Minister of Immigration, Refugees and Citizenship included the expectation that Canada would attract “more than one million new permanent residents” between 2020 and 2022 (Anonymous 2019). A total of 341000 people immigrated to Canada in 2019 (El-Assal 2020). That number was also forecast to apply to 2020 before the COVID-19 outbreak (Acres 2020). The mandate letter implies that the number would remain high, so I recalculated the expected impact on CO_2_ assuming 341000 annual immigrants through 2030 (S2 Appendix).

**References**

Acres D. 2020. Canada to welcome 341,000 new immigrants in 2020. CIC Daily 6 January 2020. <https://www.cicdaily.com/canada-to-welcome-341000-new-immigrants-in-2020/> (accessed 30 January 2020).

Anonymous. 2019. December 13, 2019 mandate letter to The Honourable Marco Mendicino, Minister of Immigration, Refugees and Citizenship. <https://pm.gc.ca/en/mandate-letters/minister-immigration-refugees-and-citizenship-mandate-letter> (accessed 30 January 2020).

El-Assal K. 2020. Canada broke another record by welcoming 341,000 immigrants in 2019. CIC News. <https://www.cicnews.com/2020/02/canada-broke-another-record-by-welcoming-341000-immigrants-in-2019-0213697.html#gs.e5hvmp> (accessed 22 August 2020).

Green D, Liu H, Ostrovsky Y and Picot G. 2016. Immigration, business ownership and employment in Canada. Statistics Canada Analytical Studies Branch Research Paper Series, Catalogue no. 11F0019M – No. 375.

Jaumotte F, Koloskova K and Saxena SC. 2016. Impact of migration on income levels in advanced economies. Spillover Notes. Washington: International Monetary Fund

McAuliffe M, Kitimbo A, Abel G, Sawyer A and Klatt J. 2020. Migration and migrants: regional dimensions and developments. In: McAuliffe M, Khadria B (eds) World Migration Report 2020. Geneva: International Organization for Migration, pp.53–122.

Our World in Data. 2019. University of Oxford. <https://ourworldindata.org/fossil-fuels#fossil-fuel-production> (downloaded 13 December 2019).

Radford J and Noe-Bustamante L. 2019. Facts on U.S. Immigrants, 2017. Washington: Pew Research Center. <https://www.pewresearch.org/hispanic/2019/06/03/facts-on-u-s-immigrants/> (downloaded 9 June 2020).

Statistics Canada. 2017. Focus on Geography Series, 2016 Census. Statistics Canada Catalogue no. 98-404-X2016001. Ottawa, Ontario. Data products, 2016 Census. <https://www12.statcan.gc.ca/census-recensement/2016/as-sa/fogs-spg/Facts-can-eng.cfm?Lang=Eng&GK=CAN&GC=01&TOPIC=7> (accessed 7 June 2020).

Statistics Canada. 2018. Statistics Canada, 2016 census of population. Statistics Canada Catalogue no. 98-400-X2016209. <https://www12.statcan.gc.ca/census-recensement/2016>/ (downloaded 9 June 2020).

UN DESA. 2019a. Migration data portal. <https://www.migrationdataportal.com/data?i=netmigrate&t=2020>; source data from the United Nations Department of Economic and Social Affairs (accessed 12 December 2019).

UN DESA. 2019b. International Migration 2019. United Nations Department of Economic and Social Affairs. <https://www.un.org/en/development/desa/population/migration/publications/wallchart/docs/MigrationStock2019_Wallchart.pdf> (accessed 18 December 2019).

UN DESA. 2019c. United Nations, Department of Economic and Social Affairs, Population Division (2019). World Population Prospects 2019, Online Edition. Rev. 1. <https://population.un.org/wpp/Download/Standard/Population/> (downloaded 30 January 2020).
