# Supplementary material for: On the effect of international human migration on nations’ abilities to attain CO2 emission-reduction targets
Source: PLoS One. 2021 Oct 4;16(10):e0258087. doi: 10.1371/journal.pone.0258087 (PMC8489703; doi:10.1371/journal.pone.0258087)
Supplement: S2 Appendix — (DOCX) [file pone.0258087.s004.docx]

**S2 Appendix: Additional Illustrations of the Impact of Migration on CO_2_ Emissions**

**Fig 2.1.** Cumulated CO_2_ emissions under the assumption that annual Canadian immigration increases to 341,000. The upper limit of each curve assumes that Canada maintains 2005 emission levels, the lower limit assumes that Canada achieves an annual reduction of 3% (= 30% reduction by 2030). Shading represents the range of possibilities for partial achievement of reduction targets.

**Fig 2.2.** Percent contribution of expected global CO_2_ emissions assuming immigration (2014 total) to Canada and the USA. The upper limit of each curve assumes that both countries maintain 2005 emission levels, the lower limit assumes that they achieve an annual reduction of 3% (= 30% reduction by 2030). Shading represents the range of possibilities for partial achievement of reduction targets.

**Fig 2.3.** Percent contribution of expected global CO_2_ emissions in the absence of immigration to Canada and the USA. The upper limit of each curve assumes that both countries maintain 2005 emission levels, the lower limit assumes that they achieve an annual reduction of 3% (= 30% reduction by 2030). Shading represents the range of possibilities for partial achievement of reduction targets.


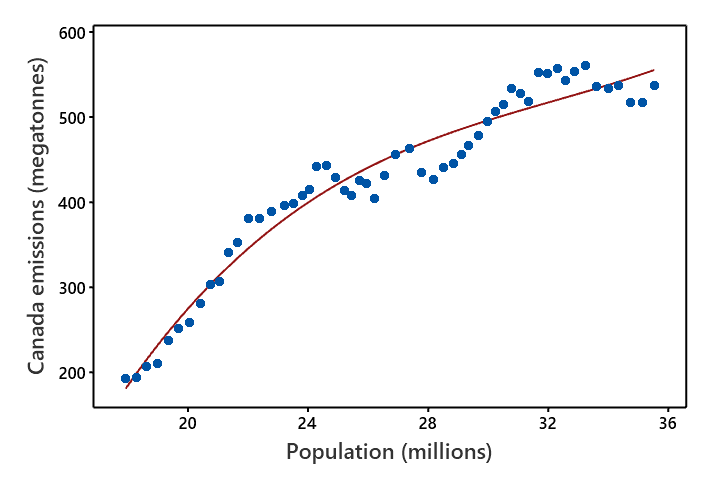


**Fig 2.4.** The cubic relationship between Canada’s CO_2_ emissions and human population size (1960-2014; *Y* = 217.0 *X* – 6.43 *X*^2^ + 0.07 *X*^3^ – 2026; *R*^2^_adj_ = 0.949; *N* =55). Line = fitted curve, dots = data.


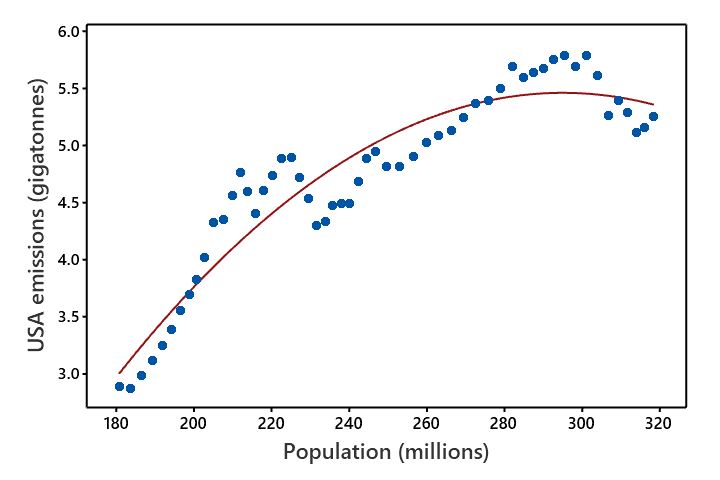


**Fig 2.5.** The quadratic relationship between USA CO_2_ emissions and human population size (1960-2014; *Y* = 0.11 *X* – 0.0002 *X*^2^ – 10.9; *R*^2^_adj_ = 0.885; *N* =55). Line = fitted curve, dots = data.
